# Structural and functional properties of the rat P2X4 purinoreceptor extracellular vestibule during gating

**DOI:** 10.3389/fncel.2014.00003

**Published:** 2014-01-29

**Authors:** Milos B. Rokic, Stanko S. Stojilkovic, Hana Zemkova

**Affiliations:** ^1^Department of Cellular and Molecular Neuroendocrinology, Institute of Physiology of the Academy of Sciences of the Czech RepublicPrague, Czech Republic; ^2^Section on Cellular Signaling, Program in Developmental Neuroscience, The Eunice Kennedy Shriver National Institute of Child Health and Human Development, National Institutes of HealthBethesda, MD, USA

**Keywords:** ATP, cadmium, gate, ion access, lateral portals, purinergic receptors

## Abstract

P2X receptors are ATP-gated cation channels consisting of three subunits that are mutually intertwined and form an upper, central, and extracellular vestibule with three lateral portals and the channel pore. Here we used cysteine and alanine scanning mutagenesis of the rat P2X4R receptor V47–V61 and K326–N338 sequences to study structural and functional properties of extracellular vestibule during gating. Cysteine mutants were used to test the accessibility of these residue side chains to cadmium during closed-open-desensitized transitions, whereas alanine mutants served as controls. This study revealed the accessibility of residues E51, T57, S59, V61, K326, and M336 to cadmium in channels undergoing a transition from a closed-to-open state and the accessibility of residues V47, G53, D331, I332, I333, T335, I337, and N338 in channels undergoing a transition from an open-to-desensitized state; residues E56 and K329 were accessible during both transitions. The effect of cadmium on channel gating was stimulatory in all reactive V47–V61 mutants and inhibitory in the majority of reactive K326–N338 mutants. The rat P2X4 receptor homology model suggests that residues affected by cadmium in the closed-to-open transition were located within the lumen of the extracellular vestibule and toward the central vestibule; however, the residues affected by cadmium in the open-to-desensitized state were located at the bottom of the vestibule near the pore. Analysis of the model assumed that there is ion access to extracellular and central vestibules through lateral ports when the channel is closed, with residues above the first transmembrane domain being predominantly responsible for ion uptake. Upon receptor activation, there is passage of ions toward the residues located on the upper region of the second transmembrane domain, followed by permeation through the gate region.

## INTRODUCTION

Providing the crystal structure of the zebrafish purinergic P2X4.1 receptor (zP2X4.1R) in its closed state (Kawate et al., 2009) was a landmark achievement that confirmed previous findings about the trimeric organization of these ATP-gated channels (Nicke et al., 1998). Each P2XR subunit is composed of two transmembrane domains (TM1 and TM2) that are separated by a large extracellular loop and N- and C-termini, which are located intracellularly (Brake et al., 1994; Valera et al., 1994). The crystallization study also confirmed that three TM2 α-helices form the P2XR pore (Rassendren et al., 1997; Egan et al., 1998), as well as a hydrophobic barrier to ion flow, called a gate, and an extracellular vestibule on the ion channel, located above the gate (Khakh and Lester, 1999). The study also revealed the existence of two additional vestibules (central and upper) and lateral fenestrations, which raised the possibility that cations travel through a central pathway that spans the entire length of the ectodomain and/or through three lateral portals that are formed at the interfaces of the adjoining subunits. Furthermore, amino acid residues that comprise the ion access portals were shown as natively unfolded regions of the zP2X4.1R molecule (Kawate et al., 2009). The subsequent crystal structure study of this receptor with and without bound ATP showed that the lateral fenestrations are encompassed by amino acid residues above the TM domains in a β-sheet conformation (Hattori and Gouaux, 2012).

These findings have prompted investigations of the pathway through which ions traverse the extracellular domain of P2XRs to enter/exit the TM pore. One study that addressed this problem suggested the importance of residues I332, T336, and T339 for forming an ion gate in the rat P2X2R (rP2X2R), and it established that the opening of the gate is accompanied by movement of the pore-lining regions, which narrow toward the cytosolic end of TM2 (Kracun et al., 2010). These conclusions are in agreement with those of earlier studies that showed the relevance of residues I328, I332, and T336 in ion gating (Stoop et al., 1999; Jiang et al., 2001; Li et al., 2008). The second study (Kawate et al., 2011) also suggested that ions access the pore by using the lateral fenestrations, which breathe as the gate opens. Their experiments raised the possibility of ions accessing the upper vestibule, which could play a regulatory function. The ion access point was also studied in the human P2X4 receptor (hP2X4R). The main conclusions of this study were that lateral portals are preferentially used because of their favorable diameters and that residues E56 and D58 are crucial for ion access to the extracellular vestibule (Samways et al., 2011).

In line with these investigations, here we focused on structural and functional properties of extracellular vestibule during gating by identifying the amino acid residues that are important for the interaction with the ion during closed-to-open and open-to-desensitized state transitions. Alanine and cysteine scanning mutagenesis was performed on the rP2X4R extracellular vestibule region encompassing the V47–V61 and K326–N338 sequences. Because cadmium ion is widely used in screening of surface accessibility of amino acids from membrane proteins (Li et al., 2008; Kracun et al., 2010; Samways et al., 2011), but is also acting as an allosteric modulator of P2X4R (Coddou et al., 2011), here we dissected the native allosteric effects of cadmium ion from cysteine binding effects. In addition, a homology model of rP2X4R in closed and open states was done to discuss topological characteristics of cadmium-sensitive mutants and to propose a model of ion access and conformational changes of extracellular vestibule during gating.

## MATERIALS AND METHODS

### CELL CULTURE AND TRANSFECTION

Recombinant rP2X4R channels were expressed in human embryonic kidney 293T cells (American Type Culture Collection, Rockville, MD, USA), which were grown in Dulbecco’s Modified Eagle Medium supplemented with 10% fetal bovine serum, 50 U/ml penicillin, and 50 μg/ml streptomycin. Cells were grown in a humidified 5% CO_2_ atmosphere at 37°C. Transfection was performed using the jetPRIME TM polymer-based transfection reagent, according to the manufacturer’s instructions (PolyPlus-transfection, Illkirch, France).

### DNA CONSTRUCTS

Complementary DNA sequences of wild type (WT) rP2X4R were subcloned into the pIRES2-EGFP vector (Clontech, Mountain View, CA, USA). To generate the mutants, oligonucleotides (synthesized by VBC-Genomics, Vienna, Austria and Sigma Chemical Company, USA) that contained specific point mutations were introduced into the rP2X4/pIRES2-EGFP template by using PfU Ultra DNA polymerase (Agilent Technologies Inc., USA). To isolate the plasmids for transfection, a High-Speed Plasmid Mini Kit (Geneaid, Shijr, Taipei, Taiwan) was used. Dye terminator cycle sequencing (ABI PRISM 3100, Applied Biosystems, Foster City, CA, USA) was used to identify and verify the mutagenesis outcomes. The sequencing was performed by the DNA Sequencing Laboratory, Institute of Microbiology, ASCR, Prague.

### PATCH CLAMP RECORDINGS

ATP-induced whole-cell currents were recorded at -60 mV using an Axopatch 200B patch-clamp amplifier (Axon Instruments, Union City, CA, USA). The recordings were captured and stored using the Digidata 1322A and pCLAMP9 software. The cell culture was perfused with an extracellular solution that contained the following: 142 mM NaCl, 3 mM KCl, 2 mM CaCl_2_, 1 mM MgCl_2_, 10 mM HEPES, and 10 mM D-Glucose, adjusted to pH 7.3 with 1 M NaOH. The patch electrodes were filled with a solution containing the following: 154 mM CsCl, 11 mM EGTA, and 10 mM HEPES, adjusted to pH 7.2 with 1.6 M CsOH. The control and ATP-containing solutions were applied via a perfusion system (RSC-200, BIOLOGIC, Claix, France).

### EXPERIMENTAL PROTOCOLS

To probe the surface accessibility of particular amino acid residues within the extracellular vestibule region of rP2X4R, 20 μM cadmium was applied, a concentration that was based on previous efficacy reports involving this cadmium concentration in experiments with rP2X2R (Kracun et al., 2010) and hP2X4R (Samways et al., 2011). Two experimental protocols were used in our study. Protocol 1: cadmium was applied for 1 min, followed by a transient (2 s) application of ATP in the presence of cadmium. Because allosteric binding sites for cadmium ions are believed to be numerous and could stimulate and/or inhibit the channel activity (Coddou et al., 2011), ATP was applied at the EC_50_ concentration to permit estimates of both stimulatory and inhibitory effects. Protocol 2: cadmium was perfused during the application of 100 μM ATP. Specifically, cadmium was applied at 50 ± 10% of the current rundown for 2–15 s, depending on the rate of receptor desensitization. The effect of cadmium was measured as the change in current amplitude (in %) immediately after ion application compared with the current amplitude before cadmium application. The same application protocols have been used for measuring cadmium effects on the WT receptor and alanine and cysteine mutants. Only the first (naïve) response was considered to exclude the impact of receptor internalization on gating (Bobanovic et al., 2002; Royle et al., 2002, 2005).

### HOMOLOGY MODELING

**FIGURE 1:**
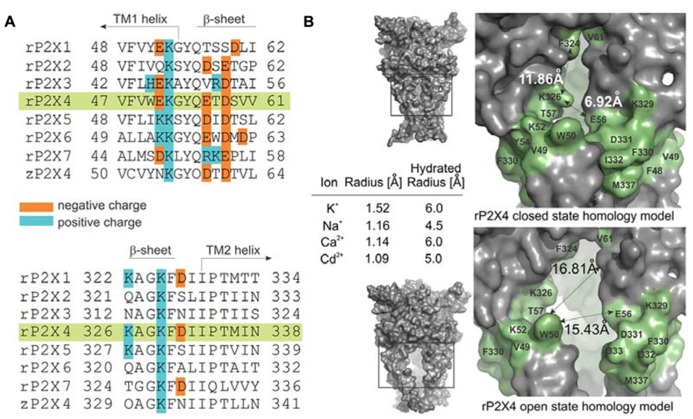
**Characteristics of the ion access point of P2XRs. (A)** Multiple sequence alignment of seven rat P2XRs compared to zP2X4.1R. The sequence alignments contain the amino acid residues that are homologous to the V47–V61 and K326–N338 segments of rP2X4R (green shade), and the positively and negatively charged residues are indicated. **(B)** The surface landscape of the homology model for rP2X4R in a closed and open state; the entire molecule (left panels) and the selected regions (right panels). The residues comprising the peptide segments from V47–V61 and K326–N338 are presented in green; double arrows and numbers show average distances encompassing the ion entrance point. The Table within the insets summarizes the ion radii of physiologically permeable ions through P2X4R and cadmium ions in hydrated and non-hydrated states.

Because the sequence identities of rP2X4R (P51577) and zP2X4.1R (Q6NYR1) are 62.4% homologous, a homology model of the rP2X4R was developed using the automated mode of the SWISS-MODEL server (Schwede et al., 2003). A tertiary structure template was extracted from the Brookhaven Protein Data Bank under the accession number 4DW1 for the zP2X4.1R in the ATP-bound open state and 4DW0 for the receptor in the apo-closed state. Model quality was estimated by a SWISSMODEL through estimation of a QMEAN4 score, which was 0.593 (Benkert et al., 2008). All graphical representations of the protein structure were prepared using PyMOL software (DeLano Scientific LLC, USA). The rP2X4R homology models of lateral portals and extracellular vestibule in closed and open states are shown in **Figure 1B** and described in the section “Model Prediction of the Position of Residues of Interest.”

### CALCULATIONS

The concentration-response data were taken from (Rokic et al., 2013), and the ATP-induced current data points were fitted with the equation *y = I*_max_*/*[1 + (EC_50_/*x*)^h^]; *y* is the amplitude of the ATP-induced current, *I*_max_**is the maximum current amplitude induced by supramaximal doses of ATP, *h *is the Hill coefficient (fixed to 1.3 in all cases), and *x *is the concentration of ATP (SigmaPlot 2000 v9.01; SPSS Inc., Chicago, IL, USA). All numerical values in the text are reported as the mean ± SEM. Significant differences (*p* < 0.05) between means for the WT receptor, cysteine mutants, and alanine mutants were determined by an ANOVA test followed by *post hoc* *t*-test analysis with Bonferonni correction for three sets of data using SigmaStat 2000 v9.01.

## RESULTS

### EXPERIMENTAL MODEL

**Figure 1A** illustrates amino acid diversity of extracellular vestibule sequences among rat P2X subunits and different content of positively and negatively charged residues of these sequences. The low evolutionary conservation (Kaczmarek-Hajek et al., 2012) and the different content of positively and negatively charged amino acid residues of these sequences among receptor subtypes prompted us to examine the hypothesis that the mechanism of ion access is isoform-specific. As a receptor model, we selected the rP2X4R and as an expression system human embryonic kidney 293T cells. We used cysteine and alanine scanning mutagenesis of the extracellular vestibule’s V47–V61 and K326–N338 sequences; cysteine mutants were used to test the accessibility of these residue side chains to reporters during closed-open-desensitized transitions, whereas alanine mutants served to exclude the possible effects caused by mutation-induced changes in cadmium binding at native allosteric sites.

The amino acid surface accessibility reporters commonly used in P2XR research include methanethiosulfonate (MTS) reagents (Egan et al., 1998; Stoop et al., 1999; Haines et al., 2001; Jiang et al., 2001; Li et al., 2008; Roberts et al., 2009; Allsopp et al., 2011; Kawate et al., 2011), silver ion (Egan et al., 1998; Haines et al., 2001; Li et al., 2008; Jindrichova et al., 2011), and cadmium ion (Li et al., 2008; Kracun et al., 2010; Samways et al., 2011; Heymann et al., 2013). Because MTS reagents are not of the appropriate size and the molecular properties as native cations, and silver ions are photosensitive requiring the use of a chloride-free extracellular solution, we used the cadmium–cysteine interaction to study ion accessibility of the receptor vestibule. The ionic radius of a cadmium ion in its hydrated and anhydrous state is comparable to both ionic radii of physiologically gated ions passing through the rP2X4R pore (**Figure 1B**, inset; Kielland, 1937; Shannon, 1976). The permeability of P2XRs to calcium (Egan and Khakh, 2004) further suggests that the divalent property of cadmium should not represent an obstacle for studies on gating function.

### CADMIUM EFFECTS ON WT-rP2X4R

**FIGURE 2:**
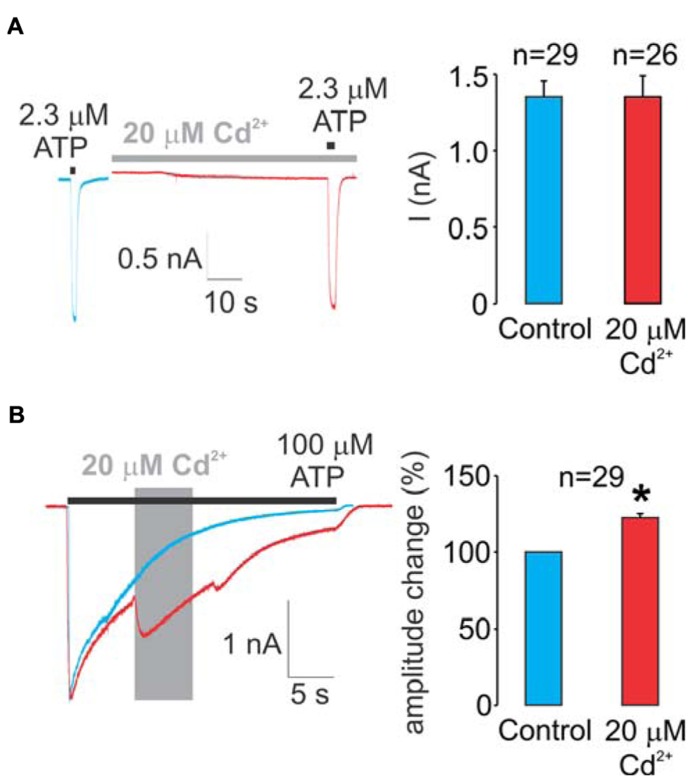
**Effects of cadmium on WT rP2X4R gating. (A)** A lack of pre-application during 1 min and a subsequent 2-s co-application of 20 μM cadmium on ATP-induced peak current response. 2.3 μM ATP (applied for 2 s) is the EC_50_ dose for the WT receptor. **(B)** Effect of the application of 20 μM cadmium ion on 100 μM ATP-induced sustained current. Cadmium was applied within 50 ± 10% desensitization decay (gray area) and significantly amplified the current amplitude, on average 22 ± 3%. Left panels show representative traces; control traces are blue, and traces with cadmium treatment are red. Right panels illustrate mean ± SEM values, with n shown above bars; * illustrates significant difference, *P* < 0.01. To avoid the impact of desensitization and the subsequent internalization on the current amplitude and patterns, control, and experimental traces were obtained in separate cells during a single ATP application.

As stated in detail in the section “Materials and Methods,” two protocols were used in our experiments. **Figure 2A** summarizes data using the protocol-1. We initially determined the ATP EC_50_ value for the WT receptor in our experimental conditions (2.3 ± 0.1 μM). Left panel shows representative traces from two different cells, control (blue trace) and a cell pretreated with cadmium for 60 s and cadmium plus ATP (red trace). These data clearly indicate a lack of cadmium influence on the peak current amplitude during the short (2 s) application of 2.3 μM ATP.

**Figure 2B** summarizes experiments with the WT receptor using protocol-2. In the absence of cadmium, the receptor desensitized almost completely during the sustained application of supramaximal (100 μM) ATP (blue trace). In contrast to protocol-1, the allosteric effect of cadmium on rP2X4R gating was visible when using protocol-2; the application of cadmium during the ATP treatment transiently reversed the process of desensitization by amplifying the current (red trace). On average, this amplification was 22 ± 3% (right panel). Note that in the presence of cadmium, the receptor continues to desensitize. Because cadmium shows allosteric effects when applied by protocol-2 but not protocol-1, all experiments with protocol-2 were performed in alanine mutants as well to distinguish between native cadmium allosteric effects and cysteine binding effects.

### CADMIUM EFFECTS ON MUTANTS

To study the accessibility of rP2X4R residue side chains to cadmium ions, we used previously generated single residue alanine (controls) and cysteine mutants of V47–V61 and K326–N338 sequences (Rokic et al., 2013). Most of mutants were functional, which is consistent with analysis conducted previously on hP2X1R (Allsopp et al., 2011), rP2X2R (Rassendren et al., 1997; Li et al., 2004; Friday and Hume, 2008; Jiang et al., 2010), and rP2X4R (Popova et al., 2010). **Table 1** lists the functional mutants. The EC_50_ (column two) and *I*_max_ values (column three) for WT and functional mutants were adopted from (Rokic et al., 2013). Column four shows mean values ± SEM of the cadmium effect on current induced by EC_50_ dose ATP during 1 min cadmium pre-application. Column five summarizes the effects of cadmium transiently applied during stimulation with 100 μM ATP for 2–15 s (protocol-2).

**Table 1:** Effect of pre-application and co-application of cadmium ion on alanine and cysteine rP2X4R mutants.

Receptor	EC_50_ (μM)	*I*_max_ (nA)	Protocol 1 (%)	Protocol 2 (%)
WT	2.3 ± 0.4	2.3 ± 0.2	100 ± 14	122 ± 3
V47A	5.2 ± 0.7	2.1 ± 0.2	–	^#^133 ± 3
V47C	2.3 ± 0.7	2.8 ± 0.5	114 ± 15	^#^199 ± 3*
F48A	2.4 ± 0.3	2.3 ± 0.2	–	^#^99 ± 1*
F48C	2.0 ± 0.9	2.9 ± 0.5	111 ± 13	^#^104 ± 1*
W50A	3.6 ± 0.3	1.9 ± 0.2	–	^#^147 ± 6*
W50C	4.4 ± 0.8	2.2 ± 0.8	100 ± 16	^#^157 ± 5*
E51A	1.6 ± 0.6	1.2 ± 0.2*	–	^#^41 ± 6*
E51C	3.5 ± 0.5	2.1 ± 0.5	150 ± 20*	^#^134 ± 5
K52A	3.1 ± 1.1	3.0 ± 0.4	–	^#^128 ± 4
K52C	2.4 ± 0.6	2.6 ± 0.4	111 ± 23	^#^132 ± 3
G53A	2.8 ± 0.6	2.9 ± 0.3	–	^#^121 ± 4
G53C	3.8 ± 0.5	2.2 ± 0.5	100 ± 16	^#^162 ± 3*
E56A	2.0 ± 0.9	1.6 ± 0.2	–	^#^134 ± 4
E56C	3.9 ± 0.7	1.9 ± 0.2	262 ± 45*	^#^222 ± 18*
T57A	1.9 ± 0.6	2.2 ± 0.3	–	^#^109 ± 2
T57C	2.0 ± 0.3	1.9 ± 0.2	162 ± 20*	^#^130 ± 3
D58A	3.2 ± 1.3	0.6 ± 0.1*	–	^#^100 ± 1*
D58C	2.3 ± 0.9	0.9 ± 0.1*	84 ± 30	^#^91 ± 2*
S59A	2.9 ± 1.3	2.6 ± 0.4	–	^#^102 ± 2*
S59C	2.0 ± 0.5	2.5 ± 0.3	220 ± 32*	^#^96 ± 1*
V60A	2.4 ± 0.6	2.3 ± 0.4	–	^#^101 ± 1*
V60C	2.1 ± 0.8	2.6 ± 0.5	116 ± 18	^#^100 ± 1*
V61A	2.9 ± 0.4	2.1 ± 0.3	–	^#^116 ± 2
V61C	3.7 ± 1.5	2.5 ± 0.3	166 ± 22*	^#^117 ± 2
%WT	2.3 ± 0.4	2.3 ± 0.2	100 ± 14	^#^122 ± 3
K326A	1.7 ± 0.6	1.9 ± 0.3	175 ± 34*	^#^126 ± 2
K326C	2.4 ± 0.6	1.3 ± 0.2		^#^129 ± 2
G328A	2.6 ± 0.9	1.9 ± 0.5	107 ± 16	^#^114 ± 2
G328C	2.7 ± 0.6	2.2 ± 0.3		^#^111 ± 4
K329A	4.9 ± 1.5	1.4 ± 0.1	43 ± 20*	^#^125 ± 3
K329C	4.0 ± 1.2	1.8 ± 0.3		^#^55 ± 4*
F330A	3.2 ± 1.2	1.5 ± 0.3	100 ± 13	^#^118 ± 2
F330C	4.0 + 1.2	0.8 ± 0.1*		^#^121 ± 2
D331A	1.5 ± 0.2	1.8 ± 0.3		^#^120 ± 2
D331C	2.1 ± 0.2	2.3 ± 0.4	100 ± 15	^#^64 ± 3*
I332A	1.3 ± 0.3	1.5 ± 0.4	110 ± 21	^#^100 ± 1*
I332C	1.6 ± 0.3	2.2 ± 0.3		^#^75 ± 2*
I333A	2.9 ± 0.8	1.6 ± 0.1	100 ± 22	^#^101 ± 1*
I333C	2.8 ± 1.1	1.9 ± 0.3		^#^196 ± 15*
P334A	1.0 ± 0.3*	1.3 ± 0.3	100 ± 35	^#^101 ± 1*
P334C	1.4 ± 0.4	0.6 ± 0.1*		^#^105 ± 1*
T335A	1.8 ± 0.6	2.2 ± 0.2	110 ± 21	^#^121 ± 2
T335C	2.6 ± 0.5	1.8 ± 0.2		^#^73 ± 2*
M336A	2.5 ± 0.3	2.2 ± 0.5	150 ± 20*	^#^104 ± 2*
M336C	1.2 ± 0.2	2.3 ± 0.5		^#^110 ± 1
I337A	2.9 ± 1.3	2.0 ± 0.4	100 ± 31	^#^128 ± 3
I337C	4.1 ± 0.6	1.0 ± 0.2*		^#^68 ± 5*
N338A	2.0 ± 0.2	3.5 ± 0.4*	100 ± 25	^#^125 ± 3
N338C	1.1 ± 0.2	3.2 ± 0.5*		^#^155 ± 4*

The data are expressed as the mean ± SEM from 12 to 40 measurements per mutant and 96 measurements for the wild type (WT) receptor. The statistical significance was estimated by ANOVA followed by *post hoc* two-way *t*-test with Bonferroni correction, comparing the level of cadmium effect between WT and mutant receptors (*p < 0.05) and between alanine and cysteine mutants of the same residue (^#^*p* < 0.05). Fifth and fourth columns depict the percentage of ATP induced amplitude change elicited by 20 μM cadmium acquired by protocol 1 and protocol 2.

**FIGURE 3:**
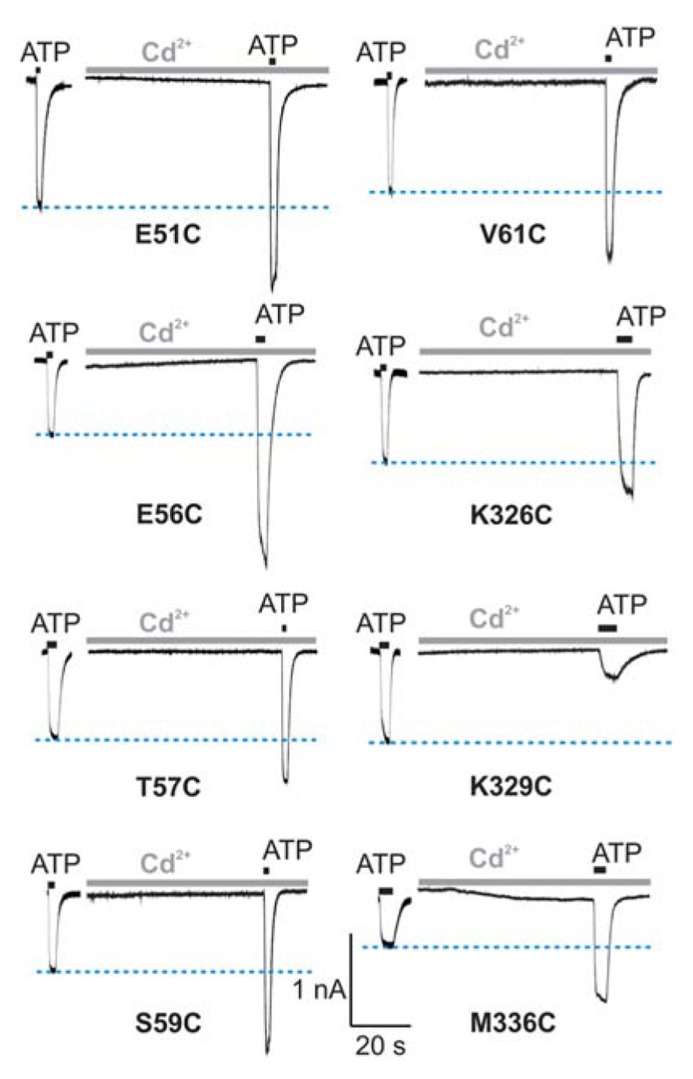
**Protocol 1: the effect of cadmium ion pre-application on peak amplitude of current response by mutant rP2X4Rs.** The whole-cell current traces represent the effect of 20 μM cadmium pre-applied for 1 min and subsequently co-applied with EC_50_ doses of ATP for 2 s. Horizontal blue lines represent the peak amplitude of currents in cadmium-non-treated cells. Traces shown are representative and mean ± SEM values are shown in **Table 1**. The holding potential was -60 mV.

In contrast to the WT receptor (**Figure 2A**), protocol-1 revealed that a cadmium pre-application resulted in a statistically significant current amplitude potentiating effect on the EC_50_ dose pulse of ATP for the following mutants: E51C, E56C, T57C, S59C, V61C, K326C, and M336C, whereas the K329C mutant was significantly inhibited by cadmium. **Figure 3** shows representative traces for these mutants, and **Table 1** (column four) shows mean ± SEM values.

Cadmium application during 100 μM ATP treatment (protocol-2) revealed four types of responses when compared to the WT receptor: (i) we observed statistically significant current augmentation in six mutants: V47C, W50C, G53C, E56C, I333C, and N338C; (ii) five mutants responded to cadmium application with the inhibition of current: K329C, D331C, I332C, T335C, and I337C; (iii) the effect of cadmium was lost in five mutants: F48C, D58C, S59C, V60C, and P334C mutants; (iv) the residual mutants responded with the facilitation of current, which was not significantly different from that observed in cells expressing the WT receptor (**Table 1**).

The alanine scanning mutagenesis also revealed that the stimulatory effect of cadmium was lost in eight mutants: F48A, D58A, S59A, V60A, I332A, I333A, P334A, and M336A. The cadmium response was significantly amplified in the W50A mutant, whereas the sustained current was significantly inhibited in the E51A mutant (**Table 1**). In the remaining mutants, the stimulatory effect of cadmium was comparable with the WT receptor. The same direction of changes in alanine and cysteine mutants for F48, W50, D58, S59, V60, and P334 residue mutants suggests that cysteine mutants should not be considered as cadmium hits. Hereafter, the following residues are considered as directly affected by cadmium: V47, G53, E56, K329, D331, I332, I333, T335, I337, and N338.

**FIGURE 4:**
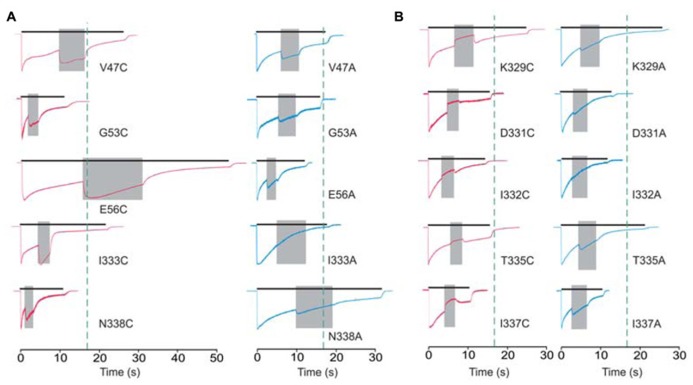
**Protocol 2: the effect of cadmium on sustained current response by mutant rP2X4Rs**. The whole-cell current recordings from -60 mV represent the effects of 20 μM cadmium transiently co-applied (gray field) with 100 μM ATP. Cadmium application was initiated at 50 ± 10% of current desensitization decay. **(A)** Enhancement of cadmium effects in cysteine mutants (red traces) and the corresponding alanine mutants (blue traces). **(B)** A transient inhibition of sustained current by cadmium (red traces). Right panels show the response of the corresponding alanine mutants. Vertical dotted lines illustrate the duration of ATP application in cells expressing the WT receptor. Traces shown are representative and mean ± SEM values for these mutants are shown in **Table 1**.

The cadmium-hit residues exhibited two types of responses: stimulation or inhibition of sustained current. **Figure 4** shows the example traces for cysteine mutants exhibiting facilitation (**Figure 4A**, left) and inhibition (**Figure 4B**, left) of current by cadmium as well as the pattern of response by the corresponding alanine mutants (**Figure 4B**, right). Note that all (three of three) affected V47–V61 mutants exhibited facilitation, and the majority of K326–N338 mutants (five of seven) exhibited inhibition of the sustained ATP-induced current. **Figure 4** also illustrates that single residue mutations affect the rates of receptor desensitization, an issue that we have not further addressed. Among mutant receptors, there was no significant correlation between the EC_50_ values for ATP and cadmium potentiation/inhibition effects (*R* = 0.48, *p* > 0.05).

### MODEL PREDICTION OF THE POSITION OF RESIDUES OF INTEREST

**FIGURE 5:**
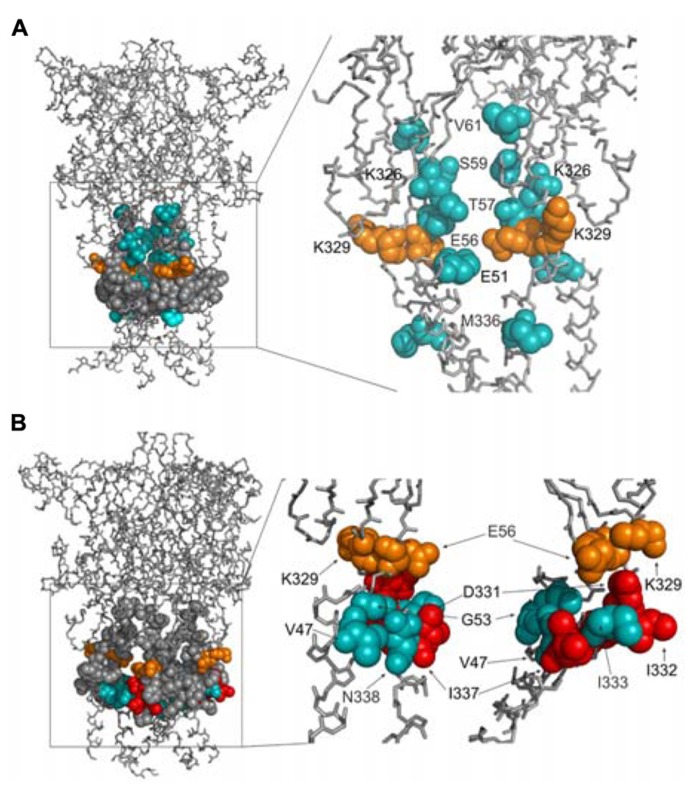
**Topology of cadmium-affected residues in the homology model of rP2X4R.** Left panel represents a wire frame model of the rP2X4R polypeptide backbone with residues from V47–V61 and K326–N338 (spheres) in closed **(A)** and open **(B)** state. Orange spheres depict the residues in cysteine mutants that were affected by cadmium in both states. Cadmium-inhibited residue mutants are depicted by red spheres and potentiated residue mutants are depicted by blue spheres. On the right panels the front subunit was removed for better visibility of the vestibule interior.

We develop the rP2X4R homology model (see Homology Modeling) to identify the position of residues of the extracellular vestibule in open and closed states. Amino acid residues that have side chains pointing toward the central cavity of the extracellular vestibule when the channel is closed are V47, G53, Q55, T57, D58, S59, V60, V61, K326, A327, G328, P334, I337, and N338. The side chains of F48, V49, W50, K52, Y54, K329, F330, I332, and M336 are pointed exclusively toward the water environment and away from the central axis of the receptor channel. Amino acid residues that could be found at the interface between the interior of the vestibule and the outside include E51, E56, D331, I333, and T335, which comprise the inverted cone-shaped access portal of the receptor and the upper segment of the pore. With exception of D58, all negatively charged residues of the rP2X4R homology model are found within the structure of the portal. The Van der Waals distances between particular extracellular vestibule residues in closed state were found to range between 6.9 and 15 Å (**Figure 1B**, top panel). The closed rP2X4R channel homology model was used to identify residues detected in experiments with protocol-1; residues affected are situated along the central axis of the vestibule (**Figure 5A**).

In contrast to the closed state, the homology model in the open state reveals slightly different distribution patterns of amino acid side chains that are found on the level of the lateral portal. The E51 residue side chain points toward the interior of the vestibule in open state, while the adjacent K52 residue points from the outside toward the level between compartments. The Van der Waals distances between particular extracellular vestibule residues in open state were found to range between 16 and 21.7 Å (**Figure 1B**, bottom panel). The open rP2X4R channel homology model was used to identify residues detected in experiments with protocol-2; the residue affected situated at the bottom of the vestibule near pore forming region as an inverted cone-shaped structure (**Figure 5B**).

**FIGURE 6:**
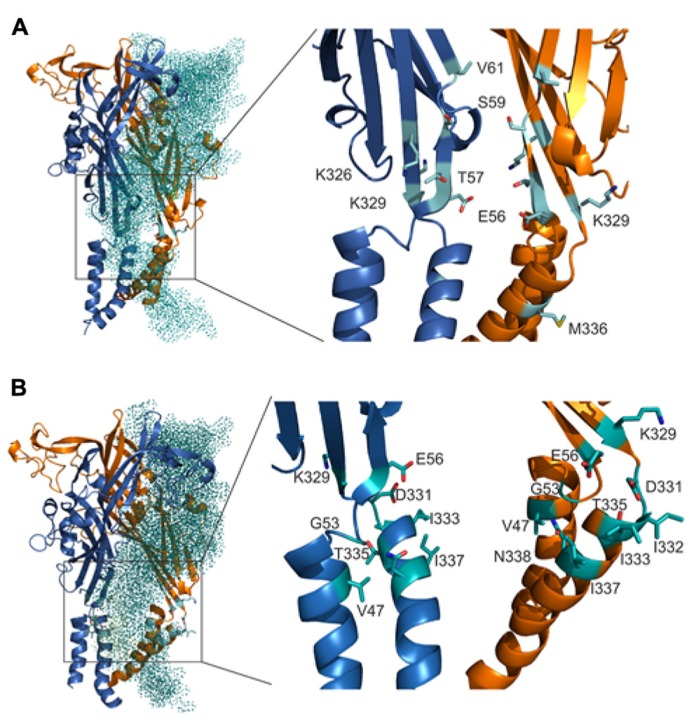
**Orientation of the side chain of residues are important for interaction with ions during gating.** The positions of cadmium-affected residues are in closed **(A)** and open **(B)** states. In the left panels, the teal colored dotted subunit was removed for better visibility of the vestibule interior. Cadmium-affected residues in rP2X4R the homology model in closed and open states are presented in the right panels. Particular cadmium hits are presented as light blue sticks on light blue ribbon segments, and different subunits are presented in orange and dark blue.

Homology model of rP2X4R in open and closed state together with cadmium accessibility data has given us an insight on how the ion accesses the extracellular vestibule. Amino acid residues that were predominantly affected during a closed-to-open state transition by cadmium were identified with their side chains pointed toward the central cavity of the extracellular vestibule (T57, K326), and on the level between the extracellular and central vestibules (S59 and V61; **Figure 6A**). M336 was the only residue found outside the central cavity with its side chain facing the extracellular environment on the level of the water-lipid interface (**Figure 6A**). This suggests that the ion uptake is facilitated by peptide segments above TM1 and that ion can pass to the central vestibule before receptor activation. All affected residues during open-desensitized state transition, with the exception of K329, T335, and I332, point their amino acid side chains towards the lumen of the vestibule (**Figure 6B**) indicating that upon ATP binding ion gets channeled toward the upper part of TM2. The K329 and E56 residues are cadmium reactive by both protocols, which indicates their role in an interaction with ions during the transition from closed-to-open-to-desensitized state. These residues are found in close proximity to each other at the entrance point to the vestibule, however, both open and closed states of our rP2X4R homology model and zP2X4.1R do not show the formation of a salt bridge (**Figures 6A,B**).

## DISCUSSION

This study focuses on the ion accessibility of amino acid residues from the extracellular vestibule of rP2X4R in a closed and open state. As a reporter, we used cadmium ions for reasons stated in the section “Experimental Model.” However, cadmium is not an ideal reporter for rP2X4R because it acts as an allosteric modulator of this receptor (Coddou et al., 2005); the extracellular zinc-binding histidine residues appears to serve as cadmium allosteric sites at P2XRs (Coddou et al., 2005; Lorca et al., 2005; Acuna-Castillo et al., 2007). We also observed the facilitatory effect of cadmium on an ATP-induced current when this metal was applied during the ATP pulse. However, this effect was observed in cells treated with 100 μM ATP, a supramaximal ATP concentration for rP2X4R, suggesting that no leftward shift in the concentration response could account for the observed effect. Thus, the most probable explanation for the results shown in **Figure 2B** is that cadmium increases the probability of the open state in the channels.

In general, the substitution of a native residue with cysteine generates an additional allosteric binding site for cadmium if the residue is accessible to this ion (Huber and Freisinger, 2013; Kunihiro et al., 2013). This in turn may or may not affect the ATP-induced current, depending on the position of the residue and protocol used for cadmium application. The affected residue could amplify or attenuate the current, and both effects indicate that the residue is accessible to cadmium, i.e., it represents the cadmium-sensitive hit. Cadmium dissociation kinetics was not studied in P2XRs, but it was addressed in relation to calmodulin and calmodulin fragments and the rate of dissociation was found to be 445 s^-^^1^ or faster (Martin et al., 1986). However, the kinetics of cadmium alteration of receptor gating was studied in WT and mutant rP2X2Rs and it has been found that cadmium modifies the receptor gating at the rates ranging from 10 to 10^5^ M^-^^1^s^-^^1^ (Kracun et al., 2010).

In our experiments, two protocols were used to identify different mutants as cadmium-sensitive. Protocol 1 was designed to avoid the possible impact of the dissociation of cadmium on the ATP response; cadmium was present during 1 min pre-application and 2-s stimulation with 2.3 μM ATP, a time sufficient to initiate the transition from closed to open state and to reach the peak current response but not the decay of the current. The τ_on_ time for cadmium to modulate rP2X2R in an open state was estimated to be approximately 0.5 s (Kracun et al., 2010), which further indicated that a fraction of channels were in an open state for a sufficient time to bind cadmium. Thus, protocol-1 reflects cadmium binding to engineered cysteine residues when the receptor is undergoing a transition from a closed-to-open state. In contrast, protocol-2 clearly detected the residues that are accessible to the ion when the channel is transiting from an open-to-desensitized state.

Protocol-1 revealed that E51, E56, T57, S59, V61, K326, K329, and M336 cysteine mutants have substantial reactivity to cadmium, whereas protocol-2 identified V47, G53, E56, K329, D331, I332, I333, T335, I337, and N338 cysteine mutants as cadmium-sensitive. Thus, out of the 16 cadmium-sensitive mutants that we observed, only E56C and K329C mutants responded to cadmium application in both protocols. These results clearly indicate different positions (conformation) of the majority of residues during exposure to cadmium in the two protocols. It is also interesting that combined experiments with two protocols suggest that all seven cadmium sensitive V47–V61 mutants show stimulatory cadmium effects, whereas five out of nine K326–N338 affected mutants show inhibitory cadmium effects, suggesting that two segments may play opposite roles in the control of closed-to-open transition.

We have developed the rP2X4R homology model and utilized the model together with available crystal structures to propose a putative ion access mechanism for the rP2X4R pore, while considering current and previous findings of amino acid side chain accessibilities in other P2XRs and the limitations of cadmium accessibility screening imposed by allosterism. Amino acid residues that were affected by cadmium during close-to-open transition were identified within the lumen of the extracellular vestibule and between the extracellular and central vestibule. Amino acid residues affected by cadmium during open to desensitized state transition were identified as the inverted cone shaped structure at the bottom of the vestibule near the pore-forming region. These striking cadmium modification patterns indicate that upon receptor activation, the vestibules widen and the E56 residue begins interacting with the ions and direct them downwards to the gate of the receptor where they interact with D331, I332, I333, T335, I337, and N338 residues.

The topological analysis of residues affected by cadmium during closed-to-open state transition reveals the possibility of ion access to the receptor through the interaction with E51, E56, T57, S59, V61, K326, K329, and M336 residues. Because the E51A mutant shows an inhibition of ATP action by cadmium in protocol-2, we estimated that cadmium could not be used to probe the accessibility of this residue. However, E51 plays a role in calcium permeability (Samways and Egan, 2007), and therefore, the accessibility of this residue in an open state is substantial. Polar non-charged residues T57 and S59 could interact with cadmium through the coordination of cadmium ions by their hydroxyl groups. The role of the hydrophobic V61 residue in ion access is still unclear, but the S59 and V61 residues are found between the middle and extracellular vestibule, which clearly confirms that the ions can access the middle vestibule. Because the channel vestibules can be charged with ions before opening, they may have a role as ion reservoirs that contribute to fast activation kinetics, which was previously described (Yan et al., 2006). The M336 residue, which is crucial for ethanol binding by the rP2X4R (Popova et al., 2010), is found at the lipid-water interface, and its interaction with cadmium in a closed state may affect receptor activation.

The K329 and E56 residues are cadmium-reactive in both protocols, which suggest roles in interactions with ions during a closed-to-open transition. Our data showed prolonged desensitization rates for both residues, which implies that they are functionally coupled. These residues are found in close proximity to each other, but both open and closed states of our rP2X4R homology model and the zP2X4.1R do not show the formation of salt bridges. Notably, most of cadmium hits for probing the open state were found below the K329 and E56 residues along the central channel axis, while most hits found in a predominantly closed state were situated above the K329 and E56 residues. However, the presence and role for a number of positively charged amino acid residues within this region remains unclear because the K52, K326, and K329 residues presented different accessibility potential to cadmium by the protocols that were used. These positively charged residues may play a role in depleting anions from the cation hydration sphere and thus facilitate cation permeability, but their exclusive role as selectivity filters has not been elucidated. Finally, the most prominent facilitatory effect of cadmium was identified in I333 and V47 mutants and our homology model has predicted a spatial proximity of these residues (not shown). This issue was also addressed in rP2X2R where V48 and I328 interaction stabilizes the closed state and facilitates lipid intercalation during channel gating (Rothwell et al., 2014).

**FIGURE 7:**
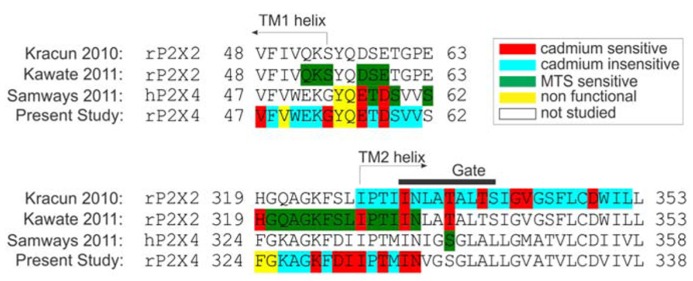
**Amino acid sequence alignment of P2XRs used in studies for probing surface accessibility of TM and ectodomain residue side chains in channels in an open state.** The alignment represents the comparison of amino acid side chain modifications with cysteine modifying MTS agents (Kawate et al., 2011; Samways et al., 2011) or cadmium (Kracun et al., 2010; Samways et al., 2011) during co-application with supramaximal doses of ATP. The gate region is presented with a black horizontal line above the TM2 domain [derived from (Kracun et al., 2010), while TM helices are marked with arrows. Non-functional rP2X4R mutants were described in Rokic et al. (2013)].

**Figure 7** summarizes a comparison of our findings from three major studies in this field. The focus in a study with rP2X2R was the I328–L353 sequence, which covered the TM2 region and accompanied five ectodomain residues (Kracun et al., 2010). It was observed that the rP2X2-T336C and I332C mutants had gating modification rates that were the same order of magnitude when cadmium was pre-applied or co-applied, which implies that these residues are fully accessible to cadmium in open and closed states. In contrast to this result, most of the TM2 residue mutants that line the permeation pathway of rP2X2R exhibited a different order of magnitude in the modification rate during the pre-application and co-application of cadmium, which suggests that the residues are accessible only during the open state. This study also helped in defining the gating rP2X2R region (indicated by the horizontal black line in **Figure 7**). Within the gating region, the I332C mutant is identified as cadmium-sensitive, which was confirmed later by (Kawate et al., 2011). We also identified the corresponding rP2X4R mutant (I337C) as cadmium hit; however we could not confirm its accessibility when channel transits from closed-to-open state. The homology model reveals a tight packing of this residue side chain triplet in the rP2X4R, and the possible formation of short distance hydrogen bonds between engineered sulfhydryl side chains at the 337 position (Rajagopal and Vishveshwara, 2005). This indicates that I337 plays a role as a hydrophobic plug.

In our study, but not in other studies (Kracun et al., 2010; Kawate et al., 2011), the N338 mutant was also cadmium-sensitive in an open state. In rP2X2R, the co-application of MTS also affected cysteine substitutions at H319 and I328 residues; the later was also detected in our experiments as a cadmium-sensitive hit. In contrast to rP2X2R, we also identified the K329C, D331C, I332C, and T335C mutants as cadmium-sensitive during the open-to-desensitized transition. Experiments with hP2X4R showed the accessibility of E56C and D58C mutants to cadmium ions during co-application. Furthermore, single channel experiments have shown that these residues do not function as selection filters (Samways and Egan, 2007). In our study, cadmium has shown insignificant effect on both D58C and D58A mutants revealing that this residue does not play a role during gating. Experiments on P2X2R that employed MTS reagents showed no effect of these reagents on analogous residues D57 and E59 (Kawate et al., 2011).

In conclusion, our findings indicate the possibility of ion entry in extracellular vestibule though lateral portals while the rP2X4R channel is closed. We also identified amino acid residues of the extracellular vestibule that interact with ion during closed-open-desensitization transition. The residues above TM1 are predominantly responsible for ion uptake into the extracellular vestibule lumen, whereas TM2 predominantly facilitates access to gate and permeation. These findings provide further insight on rP2X4R gating, which is helpful in understanding common, receptor-specific, and species-specific functions of extracellular vestibule.

## Conflict of Interest Statement

The authors declare that the research was conducted in the absence of any commercial or financial relationships that could be construed as a potential conflict of interest.
